# Performance characteristics of the digital uMI550 PET/CT system according to the NEMA NU2-2018 standard

**DOI:** 10.1186/s40658-020-00315-w

**Published:** 2020-06-26

**Authors:** Shuguang Chen, Pengcheng Hu, Yushen Gu, Haojun Yu, Hongcheng Shi

**Affiliations:** Zhongshan Hospital, Fudan University, Shanghai, China

**Keywords:** NEMA NU2-2018, Performance evaluation, uMI550 PET/CT, Silicon photomultiplier (SiPM)

## Abstract

**Background:**

The aim of this study is to conduct physical performance evaluation on the uMI550 whole-body PET/CT system according to the NEMA NU2-2018 standard.

**Methods:**

According to the NEMA NU2-2018, spatial resolution, sensitivity, scatter fraction, count-rate performance, accuracy of count losses and random corrections, image quality, and timing resolution were evaluated. Spatial resolution was measured by using a ^22^Na point source. System sensitivity was measured by inserting an ^18^F line-source in six concentric aluminum sleeves with varying diameters. Scatter fraction, count-rate performance, accuracy of count loss, and timing resolution were all calculated by analyzing dynamically acquired data of an ^18^F line-source inside a polyethylene cylinder in 20 cm diameter and 70 cm length. Image quality was assessed using a NEMA IEC body phantom with a 4:1 ratio of activity concentration of spheres to the warm background. Additionally, three patient studies were performed, with one brain scan and two whole-body scans, separately. The patient images were evaluated to get a visual first impression of uMI550.

**Results:**

The tangential, radial, and axial spatial resolutions were measured as 2.91 mm, 2.98 mm, and 2.97 mm FWHM, respectively, at 1 cm radial offset. The total system sensitivity to line source at center was 10.24 cps/kBq. A NECR peak was measured as 124.4 kcps at 18.85 kBq/mL. The scatter fraction at NECR peak was 36.65%, and the maximum count-rate error at and below NEC peak was 1.55%. Contrast recovery coefficients were from 46.5 (10 mm) to 83.9% (37 mm). The timing resolution was measured as 372 ps at low count rate.

**Conclusion:**

NEMA NU-2 2018 testing was performed on the new SiPM-based uMI550 PET/CT system. The uMI550 shows a high-spatial resolution of less than 3 mm and a good timing resolution of 372 ps. It shows clinical significances on improving potentially diagnostic ability on small lesions.

## Background

Since 1970s, PET technology has been developed significantly and has an important role in diagnosis and evaluation of functional biology processes in humans [1, 2]. In 2000, the first integrated PET/CT system became available [3]. The lutetium oxyorthosilicate (LSO) crystals [4] advanced time-of-flight (TOF) technology to stimulate the development of commercial TOF-PET scanner [5–8]. The uMI550 PET/CT (Shanghai United Imaging Healthcare Co., Ltd.) (hereinafter referred to as uMI550) was developed by using silicon photomultiplier (SiPM)-based detectors with 16.3 mm LYSO crystals. SiPMs consist of small photosensitive cells being operated in Geiger mode in which an optical photon induces an avalanche resulting in a discrete electronic signal [9]. To date, there are four commercial SiPM-based PET/CT systems: the Philips Vereos [10], the GE Discovery MI (GE Healthcare) systems [11], the Biograph Vision PET/CT (Siemens Healthineers) [12], and uEXPLORER (United Imaging Healthcare) [13, 14], besides the uMI550. NEMA NU 2-2018 (published by the National Electrical Manufacturers Association (NEMA)) [15] allows a reproducible measurement standard for evaluating physical performance of PET systems. Particularly, it has been supplemented to standardized measurement of timing resolution [16] which is one of important characteristics of TOF-PET scanners. The purpose of this study was to evaluate uMI550 physics performance, including spatial resolution, sensitivity, scatter fraction, noise equivalent count rate (NECR), image quality, accuracy of count losses and random correction, and timing resolution. Moreover, it is helpful to have a first impression of its capability in clinical situations by performing patient scans and obtaining clinic images.

## Methods

### PET/CT system

The uMI550 is an integrated 80-slice CT scanner with a whole-body LYSO PET scanner. One detector block of the PET scanner is a 7(transaxial) × 6(axial) LYSO array of 2.76 × 2.76 × 16.3 mm^3^ crystals coupled to SensL silicon photomultiplier (SiPM) sensors. And one electronic module consists of 5(transaxial) × 14(axial) detector blocks. As a result, each module is in size of ~ 100 mm(transaxial) × 240(axial)mm, covering a 24 cm axial-field-of-view (AFOV). A total of 22 electronic modules were assembled cylindrically in the PET scanner. As a result, the PET scanner has 84 crystal rings with a 722-mm diameter. The PET transverse field-of-view (FOV) is 700 mm.

### Measurements

With the following NEMA NU 2-2018 standard, physical PET performances were measured on the uMI550 included spatial resolution, sensitivity, scatter fraction, count-rate performance, accuracy of count losses and random corrections, timing resolution, and image quality.

### Spatial resolution

NEMA NU 2-2018 standard allows to use a ^22^Na point source for spatial resolution measurement. The spatial resolution was measured in two transaxial planes: one was at the ½AFOV, the other was at the $$ \raisebox{1ex}{$1$}\!\left/ \!\raisebox{-1ex}{$8$}\right. $$AFOV. Within each transaxial plane, the resolution measurement was performed at three positions, 1 cm, 10 cm, and 20 cm vertical offsets to the center of FOV. The ^22^Na point source was in an activity of 17.4 *μ*Ci. At least 5 × 10^6^ net true counts (prompts subtracted by delayed events) were acquired for each measurement position, then the counts were Fourier rebinned (FORE). 2D filtered back projection (FBP) with a ramp filter was applied for the image reconstruction without either attenuation correction, scatter correction, spatial response modeling, or time-of-flight. The obtained image was in a 1024 × 1024 image matrix with a 0.6-mm pixel size. The axial slice thickness of image was 0.6 mm. According to NEMA NU2-2018 Section 3, at each position, the axial, radial, and tangential resolutions were measured in term of both full-width-at-half-maximum (FWHM) and full-width-at-tenth-maximum (FWTM) of point spread function (PSF) of the point source image.

### Sensitivity

A NEMA PET sensitivity phantom, consisting of five concentric aluminum sleeves of 70 cm length, was used to perform sensitivity measurement. A polyethylene tube was filled with ^18^F-FDG solution to obtain a radioactive line source. The line source was in 693 mm length, and its activity was 13.07 MBq when the scan was started. The line source was inserted into the aluminum sleeve with a minimum size and placed to be parallel to the axis of the PET scanner. The phantom was placed at two positions: the center of transaxial FOV and 10 cm radial off-center. At each position, five 5-min scans were conducted by adding successively one aluminum sleeve once previous scan is finished. Random coincidence events were subtracted from prompt by using a delayed coincidence window. NEMA system sensitivity was then calculated by following NEMA NU2-2018 Section 5.

### Scatter fraction, count losses, and randoms

A scatter phantom, 70 cm long and 20 cm diameter polyethylene cylinder, was used to measure scatter fraction and count-rate performance of the PET scanner. A 690-mm-long radio-activated line source, a polyethylene plastic tube filled with FDG, was inserted into the scatter phantom. The line source insert was at 45-mm radial offset to the center of phantom and parallel to the phantom central axis. The scatter phantom was placed in the patient bed and located at the center of FOV. Moreover, the scatter phantom was rotated such that the line source was closest to the patient bed. The line source had an activity of 513 MBq at acquisition start. The whole PET scan protocol consisted of 34 timing frames. Each timing frame was composed of two durations: the scan fulfilled and paused. For #1~#6 frames, the scan was fulfilled/paused for 2 min/8 min, respectively; 3 min/3 min for #7~#10; 5 min/5 min for #11~#15; 7 min/5 min for #16~#20; 10 min/5 min for #21~#25; 20 min/20 min for #26~#29; 27 min/27 min for #30~#34. As a result, a total of ~ 11 h was used for the whole scan protocol. During each timing frame, the acquired listmode data were on-the-fly histogrammed into prompt and delayed sinograms using SSRB (single-slice rebinning). Then, total, true, random, scatter, and noise equivalent count (NEC) rates were computed according to NEMA NU2-2018 Section 4.

### Accuracy of count losses and random corrections

For the measurement on accuracy of count loss and random corrections, the PET raw data acquired in count-rate measurement were reconstructed. Randoms were estimated by using delayed coincidence events, and scatter estimation was obtained by Monte-Carlo computation. TOF- and PSF-OSEM reconstruction algorithm was used to produce 85 image slices each of which was in a 150 × 150 (4-mm pixel size) image matrix and in a 2.85-mm thickness. No post-filter was applied. The relative count-rate error was calculated according to NEMA NU2-2018 Section 6. Five image slices at each end of AFOV were excluded for this evaluation.

### Image quality

A NEMA IEC body phantom was used to assess image quality. In the phantom, a low-density lung insert (~ 0.3 g/mL density) was placed in the center, and around the insert, there were 6 fillable spheres in different sizes (10, 13, 17, 22, 28, and 37 mm diameter). The spheres were mounted such that all sphere centers were in the same transaxial plane. The background compartment volume of this phantom was 9818 mL and filled with a ^18^F-FDG solution in a radioactivity concentration of ~ 5.3 kBq/mL. All spheres were filled with a ^18^F-FDG activity concentration four times of one in background compartment. The phantom was centered in the FOV of PET scanner. To simulate activity outside of AFOV under clinical situations, the scatter phantom used in count-rate performance measurement was placed axially next to IEC body phantom. Similarly, the line source inserted in the scatter phantom was filled with ^18^F-FDG in an activity of 115.8 MBq at acquisition start. Because a 35% bed overlap of 24 cm AFOV was used in routing clinical scans, 4.68-min duration was applied for this phantom scan to mimic a whole-body scan of 100 cm total axial imaging distance in 30 min. Three duplicated scans were performed sequentially in order to improve the reproducibility of the results according to the NEMA NU2-2018 standard. The second and third scan durations were compensated to account for the decay of ^18^F. The CT scan protocol (120 kVp tube voltage, 115 mAs exposure, 0.95 mm pitch) was used in a purpose of CT-based attenuation correction. The TOF- and PSF-OSEM algorithm with 10 subsets and 3 iterations was used to obtain 85 axial slices (slice thickness = 2.85 mm) with a matrix size of 150 × 150 (4-mm pixel size). No post-filter was applied. After the reconstruction images were obtained, the Section 7.4 of “Analysis” of NEMA NU2-2018 standard was exactly followed. For each of hot spheres, a circular region-of-interest (ROI) was drawn on its central slice. This ROI was used with a diameter equal to the sphere being measured. The average counts in ROI of each sphere (denoted as *C*_H_) was calculated with taking into account of partial pixels. Then, a total of 60 ROIs in the same sizes of the ROI for the hot sphere were drawn in the background compartment of 5 image slices: the central one, the slices with + 2 cm, + 1 cm, − 1 cm, and – 2 cm axially offsets to the central. Each of 5 slices had 12 ROIs. The average counts in each background ROI (denoted as *C*_B_) was recorded. The contrast recovery coefficients (CRC) was defined as a percent contrast of (*C*_H_/*C*_B_ − 1)/(4 − 1)× 100%, where “4” was the 4:1 ratio of the FDG concentration in spheres to the one in the background compartment. Additional 12 ROIs in 37 mm diameters were drawn throughout the background, and a circular ROI, 30 mm in diameter, was drawn in the center of the lung insert image. Using the values of average count in each ROI, CRC and the corresponding background variability were calculated for all spheres together with the relative error in the lung insert with following NEMA NU2-2018 standard Section 7.4.

### Timing resolution

The acquired data of count-rate performance measurement were utilized to measure TOF resolution according to a new method proposed in NEMA NU2-2018 standard. The timing resolution was calculated as the FWHM of the time distribution of coincident events after correction on scatter, random, and off-center position of the line source insert.

### Patient study

Three patient studies were included to provide readers with a first impression of clinical images of uMI550. Firstly, a 47-year-old female patient (160.3 cm in height and 58.2 kg in weight) diagnosed with lung carcinoma was injected with 258.3 MBq ^18^F-FDG. At 66-min post-injection, a brain PET/CT scan was performed with using an 8-min PET acquisition. The clinical routine reconstruction protocol on brain imaging was used: 3D TOF- and PSF-OSEM of 2 iterations and 20 subsets, an image size of 256 × 256 in a 300-mm FOV. A 3-mm Gaussian filter was applied. The second patient was a 49-year-old female (162.3 cm in height and 53.7 kg in weight) diagnosed with rectal carcinoma was injected with 238.3 MBq ^18^F-FDG. At 72-min post-injection, a whole-body PET/CT scan was performed with using 2.5 min/bed and 35% overlap. The clinical routine reconstruction protocol on whole-body imaging was used: 3D TOF- and PSF-OSEM algorithm of 3 iterations and 10 subsets, an image size of 150 × 150 in a 600-mm FOV. A 3-mm Gaussian filter was applied. The third patient was a 54-year old female patient (146.3 cm in height and 58.3 kg in weight, BMI = 27.2), and was injected with 264.6 MBq ^18^F-FDG. At 55-min post-injection, a whole-body PET/CT scan with the same protocol to the one for the second patient was conducted. All patient studies were approved by the medical ethics review board of Zhongshan Hospital of Fudan University, and three patients were provided written informed consent.

## Results

### NEMA measurements

#### Spatial resolution

At 1 cm radial offset, the tangential, radial, and axial spatial resolutions were measured as 2.91 mm, 2.98 mm, and 2.97 mm FWHMs, respectively; at 20 cm radial offset, the resolutions were degraded to 4.08 mm, 4.14 mm, and 3.13 mm, respectively. All values are summarized in Table 1.

**Table 1:** The measured NEMA NU-2 2018 spatial resolutions averaged between two transaxial planes

Radial distance (mm)	FWHM (mm)			FWTM (mm)		
	Tangential	Radial	Axial	Tangential	Radial	Axial
10	2.91	2.98	2.97	5.35	5.49	5.62
100	3.09	3.29	2.97	5.74	5.83	5.68
200	4.08	4.14	3.13	7.12	7.60	5.91

#### Sensitivity

**Fig. 1:**
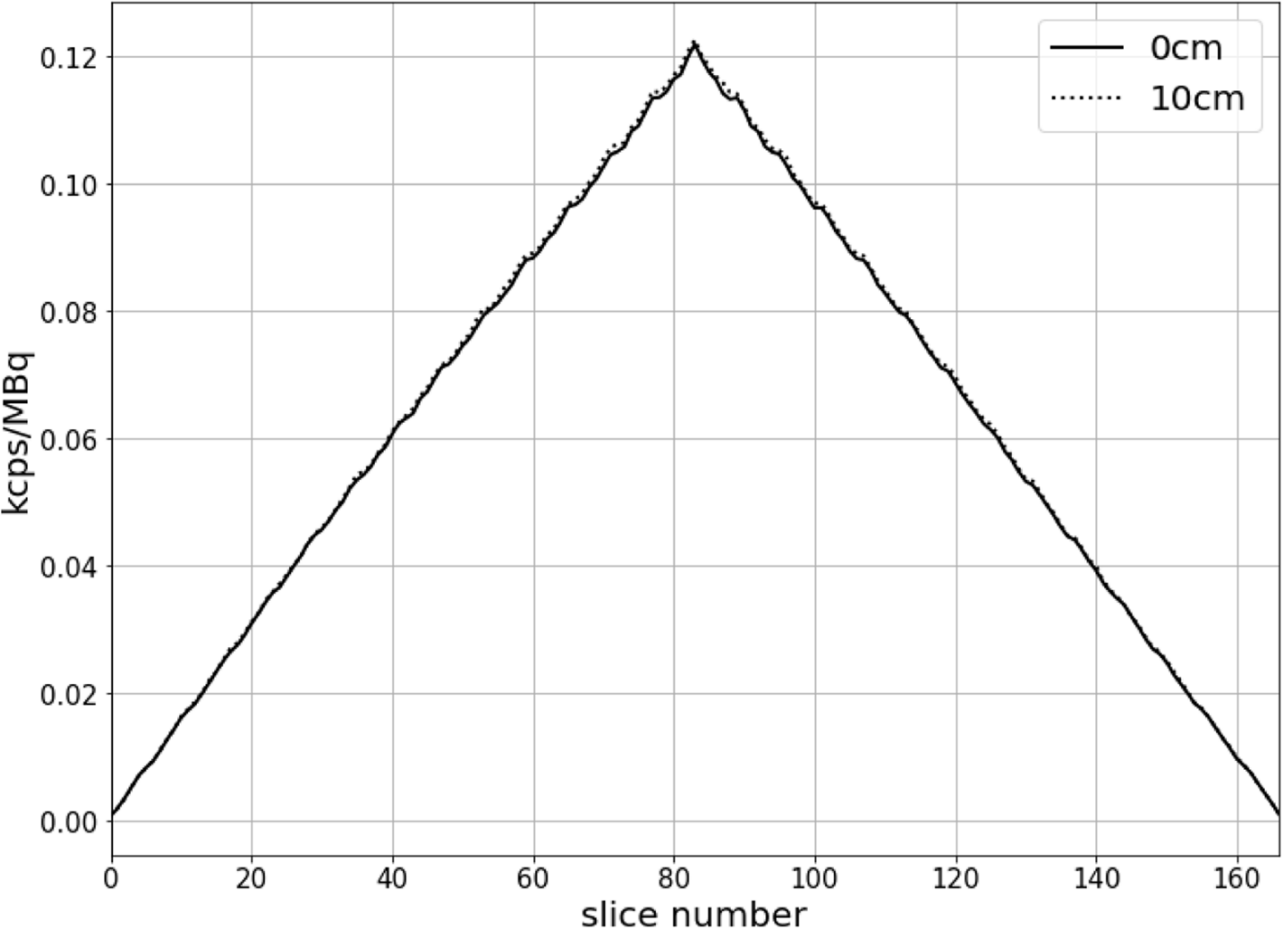
The uMI550 axial sensitivity profiles for both the 0 cm and 10 cm off-center positions

The total system sensitivity to line source at center and 10 cm radial offset were 10.24 cps/kBq and 10.32 cps/kBq, respectively. The two axial sensitivity profiles of the center and 10 cm radial offset are shown in Fig. 1.

#### Scatter fraction and count-rate performance

**Fig. 2:**
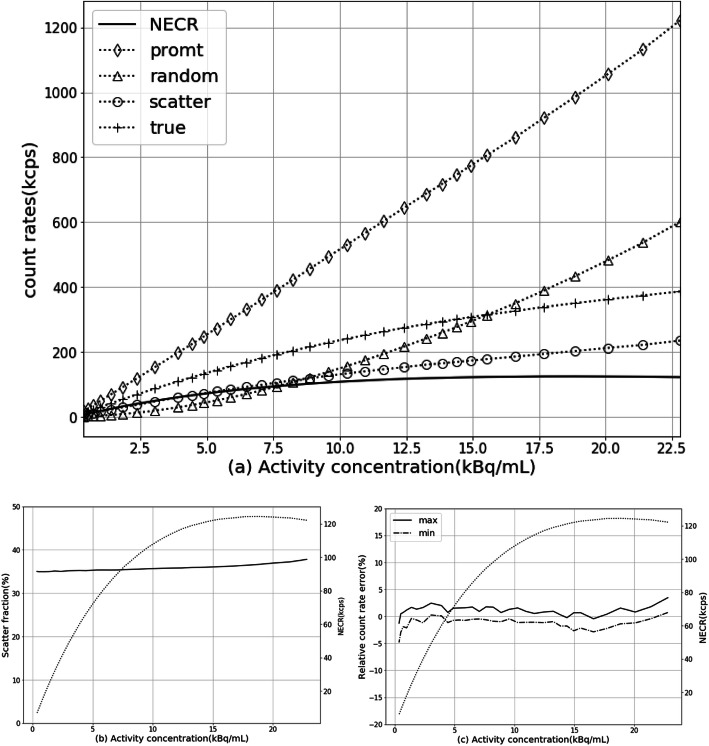
uMI550 count-rate performance. **a** The measured count-rate curves of prompt, delayed, scatter, true, and NEC rates. **b** Scatter fraction curve (solid line) in dependency on activity concentration. **c** The maximum and minimum relative count-rate error curves for difference activity radio concentration. **b**, **c** also plotted NECR curve (dotted line) by using double *Y* axes

As shown in Fig.2 a, the count rates of NEC, prompts, trues, randoms, and scatter varied with radioactivity concentration are plotted as curves, and the peak NEC rate is measured as 124.4 kcps at 18.85 kBq/mL activity concentration As shown in Fig. 2b, the scatter fraction at peak NEC rate is 36.65%. At a clinically relevant activity concentration of ~ 5.0 kBq/mL, a NEC rate of 75.7 kcps and a scatter fraction of 35.35% were observed.

#### Accuracy of count losses and random corrections

The maximum and minimum count-rate errors at different activity concentration are graphed as curves shown in Fig. 2c. The max/min count-rate relative errors at NECR peak are 1.55%/− 1.38%, respectively.

#### Image quality

**Fig. 3:**
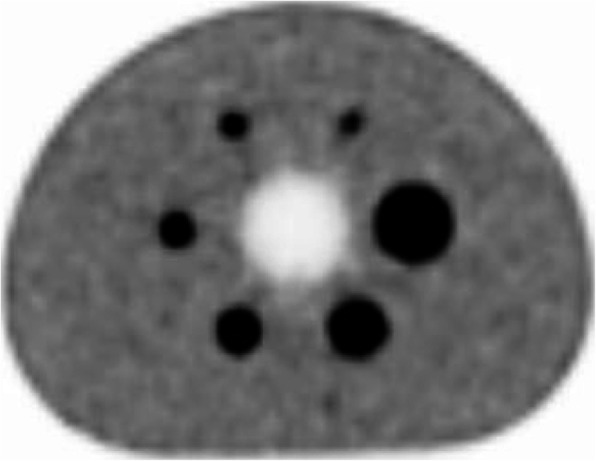
Reconstructed central image slice of IEC image quality phantom

For 4:1 ratio of sphere to background, CRC was ranged from 46.5 (10 mm) to 83.9% (37 mm) for the hot spheres. The lung residual was measured to be 3.1%. All CRCs and corresponding background variability are summarized in Table 2. The central slice of the image quality phantom for the 4:1 measurement is shown in Fig. 3.

**Table 2:** Mean-, max-, and min-percentages of contrast recovery, background variability, and lung residual for the 4:1 sphere-to-background ratio for three sequential measurements of IEC image quality phantom

Sphere diameter(mm)		10	13	17	22	28	37	Lung residual
Contrast recovery	Mean	46.5%	76.2%	79.3%	82.8%	82.1%	83.9%	
	Max	48.5%	79.5%	83.0%	85.0%	84.9%	84.5%	
	Min	43.4%	72.7%	76.4%	80.8%	80.4%	82.8%	
Background variability	Mean	6.4%	5.5%	4.7%	4.0%	3.3%	2.5%	3.1%
	Max	6.9%	6.0%	5.1%	4.5%	3.7%	2.6%	3.5%
	Min	5.7%	5.0%	4.3%	3.6%	2.9%	2.3%	2.7%

#### Timing resolution

**Fig. 4:**
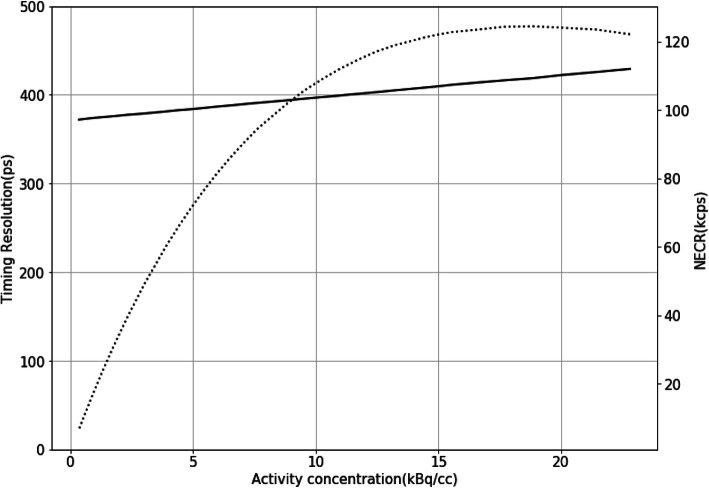
Timing resolution curve (solid line) in dependency on activity concentration and also plotted NECR curve (dotted line) by using double *Y* axes

The timing resolution of 372 ps was observed at low count-rate, and it was degraded to 419 ps at peak NECR, as shown in Fig. 4, which is worse than 372 ps at low count rate by 12.6%.

#### Patient study

**Fig. 5:**
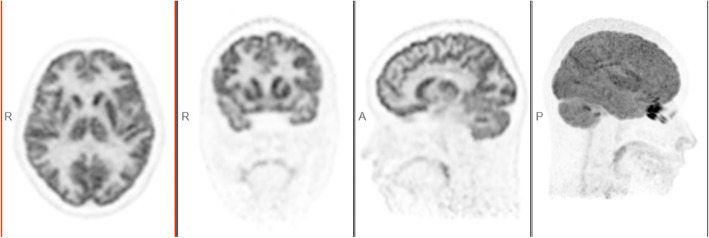
The brain image of first patient scan (from left to right): transaxial, coronal, sagittal, and MIP (maximum intensity projection). **a** MIP. **b** Lesion #1, diameter of 4.4 mm, SUVmax = 3.1. **c** Lesion #2, diameter of 5.1 mm SUVmax = 2.9

**Fig. 6:**
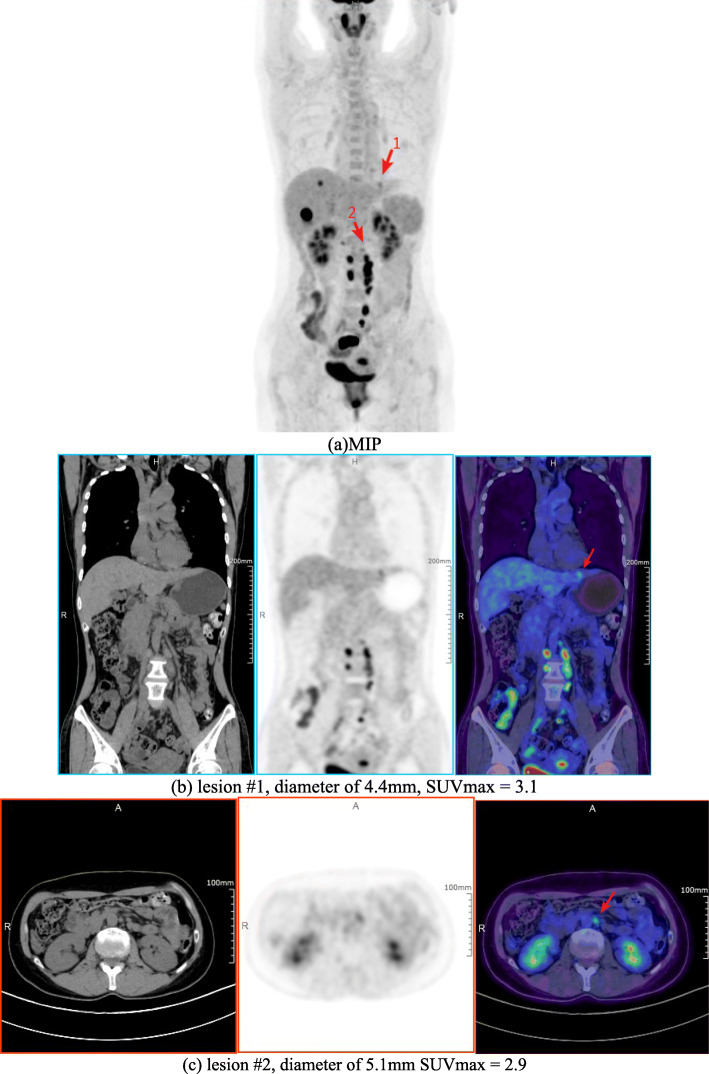
Second patient images. **a** MIP, two small lesions were pointed out by red arrows. **b** Lesion #1 images, from left to right, CT, PET, and PET/CT fusion image. **c** Lesion #2 images, from left to right, CT, PET, and PET/CT fusion image

**Fig. 7:**
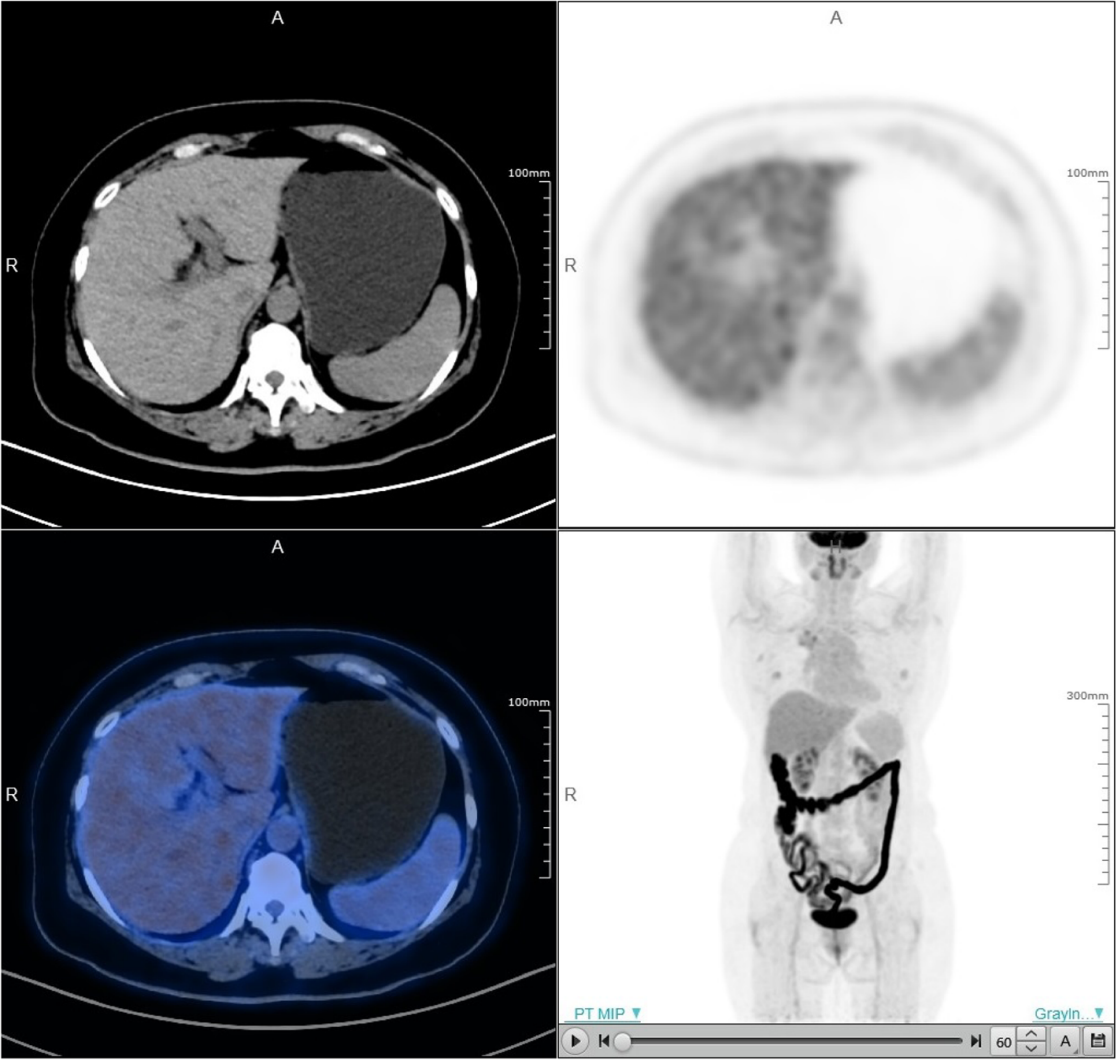
Third patient images, upper left is CT image, upper right is PET image, lower left is PET/CT fusion image, and lower right is MIP image

Figure 5 shows brain image of the first patient. The second patient images with rectal carcinoma are shown in Fig. 6. The red arrow indicates the small liver metastasis and lymphatic metastasis. Two small lesions are 4.4 mm and 5.1 mm diameters; the values of SUVmax are 3.1 and 2.9, respectively. The third patient images are shown in Fig. 7. A quite uniform liver image is obtained by the scanner.

## Discussion

According to the NEMA NU2-2018 standard, the PET scanner integrated in whole-body uMI550 PET/CT system was evaluated on the physical performances of spatial resolution, sensitivity, count-rate, accuracy of count loss, image quality, and timing resolution. These results provide references on the imaging system characteristics and comparisons with other commercial SiPM-based PET/CT scanners.

### Spatial resolution

As listed in Table 3, when compared to other LSO/LYSO + SiPM-based whole-body PET/CT systems [10–12], the uMI550 system can achieve a better spatial resolution which is mostly due to a smaller crystal size (2.76 mm) used in uMI550. From the center to 20 cm radial offset, the radial spatial resolution of the uMI550 is degraded by ~ 39%, which is smaller than others in Table 3. It could be explained due to the shorter crystal length (16.3 mm) of the uMI550 which alleviates the depth-of-interaction (DOI) effect at large radial offset although the ring of the uMI550 is in a smaller diameter (72.2 cm).

**Table 3:** The comparison of spatial resolution measurement results (FWHM) among uMI550, Siemens Biograph Vision, Philips Vereos, and GE Discovery MI (20 cm AFOV)

Radial distance (mm)	Scanner (crystal size, ring diameter, AFOV)	FWHM (mm)			
		Transverse	Tangential	Radial	Axial
10	UIH uMI550 (2.76 × 2.76 × 16.3 mm^3^, 72.2 cm, 24 cm)	2.94*	2.91	2.98	2.97
	Philips Vereos (4 × 4 × 19 mm^3^, 76.4 cm, 16.4 cm)	4.24	N.A.	N.A.	4.17
	Siemens Biograph Vision (3.2 × 3.2 × 20 mm^3^, 78 cm, 25.6 cm)	N.A.	3.6	3.5	3.5
	GE Discovery MI (3.95 × 5.3 × 25 mm^3^, 74.4 cm, 20 cm)	N.A.	3.97	4.02	4.39
100	uMI550	N.A.	3.09	3.29	2.97
	Vereos	N.A.	4.35	4.55	4.39
	Vision	N.A.	3.9	4.5	4.3
	Discovery MI	N.A.	4.23	5.28	5.63
200	uMI550	N.A.	4.08	4.14	3.13
	Vereos	N.A.	4.92	5.84	4.60
	Vision	N.A.	3.5	5.8	4.4
	Discovery MI	N.A.	4.67	7.54	5.70

*Average value of radial and tangential results

### Sensitivity

As shown in Table 4, the uMI550 has a better sensitivity performance than the Philips Vereos. It could be explained by its 24 cm AFOV which is larger than 16.4 cm one of Vereos. The comparisons in Table 4 also show both the sensitivity performances of the GE Discovery MI and Siemens Biograph Vision which are better than uMI550. Although the 24 cm AFOV of uMI550 is comparable to Vision’s 25.6 cm AFOV and even larger than Discovery MI’s 20 cm AFOV, the 20-mm/25-mm-long crystals of Vision/Discovery MI could account for enhancing sensitivity performance due to a higher scintillator’s absorption efficiency on gamma photons with longer crystals.

**Table 4:** A comparison of sensitivity performance among different scanner models. unit: cps/kBq

Radial location (cm)	uMI550	Vision	Vereos	Discovery MI (20 cm AFOV)
0	10.24	16.4	5.1	13.7
10	10.32	16.3	5.2	13.45

### Count-rate performance and accuracy of corrections

The Table 5 lists the count-rate performance measurement of uMI550 together with other three SiPM-based PET/CT system. As a result, both the NECR peak and its activity concentration of uMI550 are lower than any one of Vision, Discovery MI, and Vereos. It might be due to the large multiplexing used in uMI550 and modest sensitivity performance of the scanner. At clinical activity concentrations, i.e., at ~ 5 kBq/mL, the NECR of the uMI550 is higher than Vereos but lower than Vision or Discovery MI; this is consistent with the comparison results on sensitivity performances of the four scanners in Table 4. Lower scatter fractions of Vereos than uMI550 are observed. This might be due to 1-to-1 coupling detector design and 11.2% energy resolution of Vereos [10] in comparison with a 12% system energy resolution of uMI55 reported in its datasheet. For the measurement on accuracy of count loss and random corrections, the relative count-rate errors are better than other systems. Only a 1.5% average bias at the peak NECR of uMI550 is better than 2.9% of Vision. Moreover, the maximum count-rate errors at NECR peak are 6.8% and 3.14% for Vereos and Discovery MI, respectively, and uMI550 was only 1.55%. This might account for an appropriate correction of dead time losses and random applied on uMI550.

**Table 5:** A comparison of count-rate performance among different scanner models

	uMI550	Vision	Vereos	Discovery MI (20 cm AFOV)
NECR peak	124.4 kcps at 18.85 kBq/mL	306 kcps at 32.6 kBq/mL	153.4 kcps at 54.9 kBq/mL	193.4 kcps at 21.9 kBq/mL
Scatter fraction at NECR peak	36.65%	38.7%	33.9%	40.6%
Scatter fraction at low activity	35.35%	37%	31.7%	N/A
NECR at 5 kBq/mL	75.7 kcps	~ 120 kcps*	47.2 kcps	~ 100 kcps**

*Reading from NECR curve in (*12*) Fig. 2

**Reading from NECR curve in (*11*) Fig. 2a

### Image quality

By comparing with NEMA NU2-2012 standard [17], NEMA NU2-2018 standard requires that six spheres are all hot. The reported CRCs of spheres were obtained by using TOF- and PSF-OSEM, 150 × 150 with 4 mm pixel size and 3 iterations with 10 subsets. As a result, there is a 64% increase in CRC between the 10 mm and 13 mm spheres, from 46.5 to 76.2%. Using 6 iterations, the increase of CRC between the two spheres is 34%, from 61.9 to 83.2%. This shows a slower convergence of 10 mm sphere than 13 mm one. A similar situation can be seen for the Siemens Vision and mCT Flow, as reported in the Table 2 of the reference [12]. For mCT Flow, there was a 51% increase of CRC from 41.9% of 10 mm sphere to 63.1% of 13 mm one; conversely, for Vision, there was an 11% drop of CRC from 86.8% of 10 mm sphere to 77.2% of 13 mm sphere. Although the improved CRCs were observed by applying more iterations, the background variability was larger in the same time. Moreover, reconstruction protocols with more iterations mean more computing time that might reduce its feasibility in such clinic PET/CT centers as ours with high daily throughput. This tradeoff among CRC, background variability, and convergence suggests us that further studies on the optimization on image reconstruction parameters of uMI550, e.g., pixel size, the number of iterations and subsets, post-smoothing method, need be conducted in the future. Additionally, the newer generation of PET image reconstruction algorithms such as Bayes penalized reconstruction [18] or deep learning techniques [19] which could further address this tradeoff are also worth to be explored in the future.

### Timing resolution

The 372 ps timing resolution of uMI550 was measured by following NEMA NU2-2018 method which was proposed in [16]. With the same method, a 210-ps timing resolution of Vision was measured [12]; it is much better than uMI550. And the 372ps timing resolution of uMI550 is comparable to ones of Vereos and Discovery MI. However, the 310 ps of Vereos was measured by using ^22^Na point source [10], and the 375 ps of Discover MI was done by using a 70-cm-line source hanged in the air of FOV [11]. Time resolution measurement could be affected by some factors including the activity concentration, geometry, and position of the source [16]. A time resolution measurement using point or line source might overestimate the resolution due to a lack of sufficient scatter media similar to clinical situations of patient scans. The uMI550’s timing resolution is degraded by 12.6% from a low count rate to the peak NECR. It might be explained by detector signal pileups when the count rate goes up. And the degree of pileup is determined by multiplexing which is varied in different PET detector designs.

### Patient study

The clinical patient images were demonstrated in Figs. 5, 6, and 7. The brain image (3D TOF- and PSF-OSEM of 2 iterations and 20 subsets*,* 256 × 256 image size, 300 mm FOV) in Fig. 5 shows that uMI550 can produce high-resolution PET images. For the whole-body images (3D TOF- and PSF-OSEM algorithm of 3 iterations and 10 subsets, 150×150 image size, 600 mm FOV), as shown in Fig. 6, two small and low-contrast lesions were diagnosed in uMI550 whole-body images, and Fig. 7 shows clear contrast between muscle and subcutaneous fat. However, these clinical images should not be considered as a valid objective comparison of clinical system performance, and these images are shown only with the aim to provide readers a first impression of the clinical images acquired by the uMI550.

## Conclusion

In conclusion, NEMA NU-2 2018 testing was performed on the new SiPM-based uMI550 PET/CT systems. The uMI550 shows a high-spatial resolution of 2.95 mm/2.97 mm (transverse/axial) at 1 cm offset from the FOV center. It also shows an average sensitivity of 10.28 cps/kBq and a peak NECR of 124.4 kcps. Using LYSO crystal and SiPM-based detectors, the uMI550 provides a good timing resolution of 372 ps. The patient scan shows clinical significance of potentially improving diagnostic ability on smaller-size or lower-contrast lesions with the high-spatial resolution performance of the uMI550.

## Data Availability

All of NEMA raw data images are stored and archived on a hard disk and a CD at the Department of Nuclear Medicine, Zhongshan Hospital, Fudan University, China.

## Abbreviations

NEMA: National Electrical Manufacturers Association
^18^F-FDG: Fludeoxyglucose (^18^F)
LSO: Lutetium oxyorthosilicate
LYSO: Lutetium yttrium orthosilicate
FOV: Field-of-view
OSEM: Ordered-subset expectation maximization
TOF: Time-of-flight
SUV: Standard uptake value
PSF: Point spread function
PET/CT: Positron emission tomography/computed tomography
SiPM: Silicon photomultiplier
NECR: Noise equivalent count rate
FWHM: Full-width-at-half-maximum
FWTM: Full-width-at-tenth-maximum
FORE: Fourier rebinned
AFOV: Axial field-of-view
CRC: Contrast recovery coefficients
BMI: Body mass index
FBP: Filtered back projection
